# The most common European HINT1 neuropathy variant phenotype and its case studies

**DOI:** 10.3389/fneur.2023.1084335

**Published:** 2023-02-17

**Authors:** Marija Rozevska, Dmitrijs Rots, Linda Gailite, Ronalds Linde, Stanislavs Mironovs, Maksims Timcenko, Viktors Linovs, Dzintra Locmele, Ieva Micule, Baiba Lace, Viktorija Kenina

**Affiliations:** ^1^Medical Genetics and Prenatal Diagnostics Clinic, Children's Clinical University Hospital, Riga, Latvia; ^2^Scientific Laboratory of Molecular Genetics, Riga Stradins University, Riga, Latvia; ^3^Rare Neurological Disease Department, Pauls' Stradins University Hospital, Riga, Latvia; ^4^Radiology Department, Riga 2nd Hospital, Riga, Latvia

**Keywords:** HINT1, nerve USG, axonal polyneuropathy, population frequency, impaired mental performance

## Abstract

HINT1 is an ubiquitous homodimeric purine phosphoramidase belonging to the histidine-triad superfamily. In neurons, HINT1 stabilizes the interaction of different receptors and regulates the effects of their signaling disturbances. Changes in *HINT1* gene are associated with autosomal recessive axonal neuropathy with neuromyotonia. Aim of the study was detailed description of patients' phenotype with *HINT1* homozygous NM_005340.7: c.110G>C (p.Arg37Pro) variant. Seven homozygous and three compound heterozygous patients were recruited and evaluated using standardized tests for CMT patients, in four patients' nerve ultrasonography was performed. The median age of symptom onset was 10 years (range 1–20), with initial complaints being distal lower limb weakness with gait impairment, combined with muscle stiffness, more pronounced in the hands than in the legs and worsened by cold. Arm muscles became involved later, presenting with distal weakness and hypotrophy. Neuromyotonia was present in all reported patients and is thus a diagnostic hallmark. Electrophysiological studies demonstrated axonal polyneuropathy. Impaired mental performance was observed in six out of ten cases. In all patients with *HINT1* neuropathy, ultrasound examination showed significantly reduced muscle volume as well as spontaneous fasciculations and fibrillations. The nerve cross-sectional areas of the median and ulnar nerves were closer to the lower limits of the normal values. None of the investigated nerves had structural changes. Our findings broaden the phenotype of *HINT1*-neuropathy and have implications for diagnostics and ultrasonographic evaluation of *HINT1*-neuropathy patients.

## 1. Introduction

Loss-of-function changes in the histidine triad nucleotide-binding protein 1 (HINT1) are associated with autosomal recessive axonal neuropathy with neuromyotonia (NMAN [MIM: 137200]) (1), the symptoms of which are similar to those of Charcot-Marie-Tooth (CMT) disease. Patients usually experience their first symptoms at around 10 years of age. Patients with the *HINT1* gene variant demonstrate gradual development of motor-greater-than-sensory polyneuropathy leading to lower limb weakness and gait impairment, over time, hand muscle relaxation difficulties develop.

Nineteen variants have been causally associated with *HINT1* neuropathy in over 100 patients from Europe, North America and Asia (1–5). There are three proven founder variants in Europe (p.Arg37Pro, p.Cys84Arg and p.His112Asn) and one in China (p.Cys38Arg) (3). Among them, NM_005340.7 (*HINT1*): c.110G>C (p.Arg37Pro) is by far the most common. The allelic frequency of variant p.Arg37Pro is high in Europe, and it is absent in African American, Ashkenazi Jewish, East Asian and South Asian populations (6). There is also a certain gradient of distribution within Europe: it stems from northeast to southwest and is the most common in Eastern Europe populations, with a high carrier frequency in Central and Southeast Europe, Russia and Turkey (1, 7). However, in Latvia, the frequency of variant p.Arg37Pro is the highest known so far (see Supplementary Table 1) (8).

HINT1 is an ubiquitous homodimeric purine phosphoramidase belonging to the histidine-triad (HIT) superfamily, characterized by a conserved HIT motif (His-X-His-X-His-X-X) in the catalytic pocket. Although the mechanisms are not completely understood, the HINT1 protein is involved in the function of nervous system. In neurons, HINT1 stabilizes the interaction of different receptors and regulates the effects of their disturbances in axonal signaling. HINT1 is also known to regulate transcription factors involved in tumor progression and apoptosis (4). In the central nervous system (CNS), HINT1 interacts with the μ-opioid receptor, regulating its desensitization (4).

Although the number of *HINT1* patients diagnosed worldwide every year continues to increase, only a few homozygous cases have been described; therefore, the phenotype of *HINT1* patients has not been fully established. We performed the first systematic assessment of *HINT1* homozygous variants of p.Arg37Pro and described the full phenotype of the patients as well as a potential hot spot event in the Baltic region.

## 2. Materials and methods

### 2.1. Subjects

To assess the phenotypes of *HINT1* neuropathy patients, we analyzed 10 patients diagnosed with axonal neuropathy with neuromyotonia–2 male and 8 female—with ages ranging from 13 to 64 years. The patients were from seven families. All participating patients or their parents gave signed informed consent for participation in the study and publication of photographs.

### 2.2. Methods

All patients diagnosed with axonal neuropathy with neuromyotonia (HINT1—neuropathy) in Latvia (*n* = 10) from geneticists, neurologists and pediatric neurologists' clinical practices were recruited into this study. We extensively phenotyped and evaluated all of them.

Axonal neuropathy with neuromyotonia was diagnosed based on clinical symptoms, a neurophysiological examination and positive genetic testing. Clinical characteristics, including neurological status testing and scoring of disease severity, were evaluated by a certified neurologist using standardized tests for CMT patients, which was performed in accordance with CMT Neuropathy Score version 2 (CMTNSv2) (9). All patients underwent neurophysiological studies, including a nerve conduction study (NCS), and needle electromyography (EMG), which was performed by a certified specialist. Nerve conduction studies were performed using a Keypoint (Dantec^®^ Keypoint^®^ Focus EMG/NCS/EP System). Neuromuscular ultrasonography (NMU) was performed on 4 out of 10 patients with HINT1 neuropathy. A Cannon Aplio i900 ultrasound machine with a 22 mHz Ultra-Height frequency Hockey Stick (i22LH8) probe was used for nerve and muscle evaluation. The median and ulnar nerves were evaluated from the beginning of their peripheral part to the level of the plexus bundles, and their homogeneity and the condition of nerve fascicles were assessed in the axial and longitudinal planes. The standard protocol included examination of the *mm. interossei dorsales manus*, the *mm. lumbricales* and the *mm. hypothenar* for the presence of atrophy, fasciculations and fibrillations. For comparison, NMU was performed on patients with Charcot-Marie-Tooth type 1A (CMT1A) neuropathy, gelsolin amyloidosis neuropathy (AGel neuropathy) and amyotrophic lateral sclerosis (ALS). Additionally, all HINT1-neuropathy patients responded to an in-house survey about sociodemographic data, disease symptom onset and duration of symptoms and completed the 7-item Generalized Anxiety Disorder Scale (GAD-7) questionnaire.

The study was approved by the Central Medical Ethics Committee of Latvia (No. 3/18-03-21) and is performed in accordance with the ethical standards as laid down in the 1964 Declaration of Helsinki and its later amendments.

For the genetic analysis, research project's patients' DNA was isolated from peripheral blood. For all patients, PMP22 gene copy number changes were first excluded using the MLPA kit P405 (MRC-Holland, The Netherlands) according to the manufacturer's protocol. For individuals with a normal PMP22 copy number, exome sequencing (ES) was performed using the Twist comprehensive exome kit (Twist biosciences, USA) on a 100PE NovaSeq according to the manufacturer's protocols. The obtained raw sequencing data were mapped to the GRCh38 reference genome using BWA-mem, and SNV/indel calling was performed using DeepVariant. Mitochondrial DNA variants were called using Strelka2 with call ContinuousVf argument, and the copy number variants were identified using ExomeDepth and XHMM, as implemented in the Ximmer tool. The obtained VCFs were uploaded, annotated, and analyzed on the Illumina VariantInterpreter platform in the AD, AR, X-L, and mitochondrial modes of inheritance. Variants were classified in accordance with the ACMG variant interpretation guidelines.

For patients recruited from the Medical Genetics Clinic, Children's University Hospital of Latvia, genetic tests were performed at a private laboratory. The genomic DNA sample was randomly fragmented using non-contact, isothermal sonochemistry processing. Sequencing libraries were size-selected with a bead-based method to ensure optimal template size and amplified by polymerase chain reaction (PCR). Ready sequencing libraries were sequenced using the Illumina sequencing-by-synthesis method using paired-end sequencing (150 by 150 bases). Base called raw sequencing data were transformed into FASTQ format using Illumina software (bcl2fastq). Sequence reads of each sample were mapped to the human reference genome (GRCh37/hg19). Burrows-Wheeler Aligner (BWA-MEM) software was used for reading alignment. Duplicate read marking, local realignment around indels, base quality score recalibration and variant calling were performed using GATK algorithms (Sentieon) for nDNA. Variant data were annotated using a collection of tools (VcfAnno and VEP) with a variety of public variant databases.

## 3. Results

**Figure 1:**
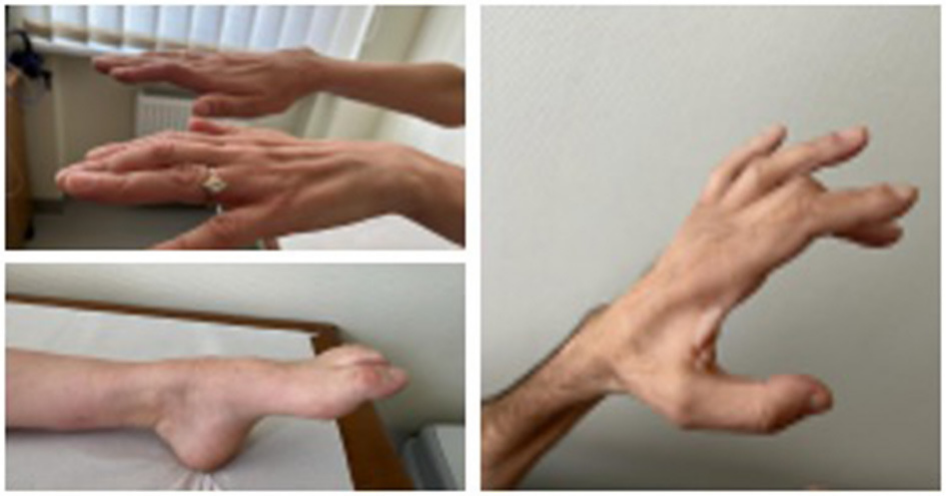
Muscle atrophy and feet deformations in patients with *HINT1* neuropathy.

To assess the phenotypes of *HINT1* neuropathy patients, we analyzed 10 patients diagnosed with axonal neuropathy with neuromyotonia. The diagnosis was confirmed by molecular analysis. Patient characteristics are described in Table 1, and photos presented in Figure 1.

**Table 1:** Characteristics of patients with HINT1—neuropathy.

**ID**	**Gender** **(male/ female)**	**Age**	**Age at the first report of symptoms**	***HINT1* genotype**	**Age at molecular testing**	**Neuromyotonic or myokymic discharges on needle EMG/clinical myotonia**	**Neurophysiological findings**	**Pes cavus/drop foot**	**Contractures**	**CMTNS**	**GAD 7 scale**
Family No. 1 ID1	F	24	10	c.110G>C, p.(Arg37Pro) homozygous	21	+ hands=legs	Axonal polyneuropathy; pure motor	+/+	+	9	13
ID2	F	18	8	c.110G>C, p.(Arg37Pro) homozygous	13	+ hands=legs	Axonal polyneuropathy; pure motor	+/+	+	10	9
Family No. 2 ID3	M	59	9	c.110G>C, p.(Arg37Pro); c.145C>T, p.(Pro49Ser)	56	+ hands>legs	Axonal polyneuropathy; motor-and-sensory	+/+	+	18	1
ID4	M	61	11	c.110G>C, p.(Arg37Pro); c.145C>T, p.(Pro49Ser)	58	+ hands>legs	Axonal polyneuropathy; motor-and-sensory	+/+	+	13	0
ID5	F	64	10	c.110G>C, p.(Arg37Pro); c.145C>T, p.(Pro49Ser)	61	+ hands>legs	Axonal polyneuropathy; motor-and-sensory	+/+	+	12	2
Family No. 3 ID6	F	35	15	c.110G>C, p.(Arg37Pro) homozygous	35	+ hands=legs	Axonal polyneuropathy; pure motor	+/+	+	9	7
Family No. 4 ID7	F	56	20	c.110G>C, p.(Arg37Pro) homozygous	56	+ hands>legs	Axonal polyneuropathy; motor-and-sensory	+/+	+	NA	12
Family No. 5 ID8	F	36	13	c.110G>C, p.(Arg37Pro) homozygous	35	+ hands>legs	Axonal polyneuropathy; motor-and-sensory	+/+	+	13	8
Family No. 6 ID9	F	13	1	c.110G>C, p.(Arg37Pro) homozygous	35	+ hands>legs	Axonal polyneuropathy; motor-and-sensory	+/+	+	7	NA
Family No. 7 ID10	F	17	4	c.110G>C, p.(Arg37Pro) homozygous	14	+ hands>legs	Axonal polyneuropathy; pure motor	+/+	+	7	6

Six out of ten patients reported symptom onset around the age of 10 years, three patients in early childhood and one in adulthood, presenting with slowly progressive muscle weakness and foot deformities. The median age of symptom onset was 10 years, ranging from 1 to 20 years. Initial complaints in all patients were distal lower limb weakness with gait impairment, combined with muscle stiffness, more pronounced in the hands than in the legs and worsened by cold. Arm muscles became involved later, presenting with distal weakness and hypotrophy. The median time from the appearance of the first symptoms to the molecular diagnosis of *HINT1* neuropathy was 27 years, ranging from 5 to 51 years.

Neurological examination in all patients revealed a steppage gait due to bilateral asymmetric foot drop, decreased or absent tendon reflexes, moderate flexion contractures of the fingers with muscle atrophy, and action neuromyotonia (more pronounced in the hands). Mild distal sensory impairment in the legs was reported in four cases. Only one of the reported patients lost ambulation due to spinal trauma following a car accident. Her symptoms of *HINT1* neuropathy overlapped with post-traumatic symptoms, and the CMTNS scale was not applicable to her evaluation. The median CMTNS score in the patient group (*n* = 9) was 10 (ranging from 7 to 18), which indicates mild neurological impairment (10).

**Figure 2:**
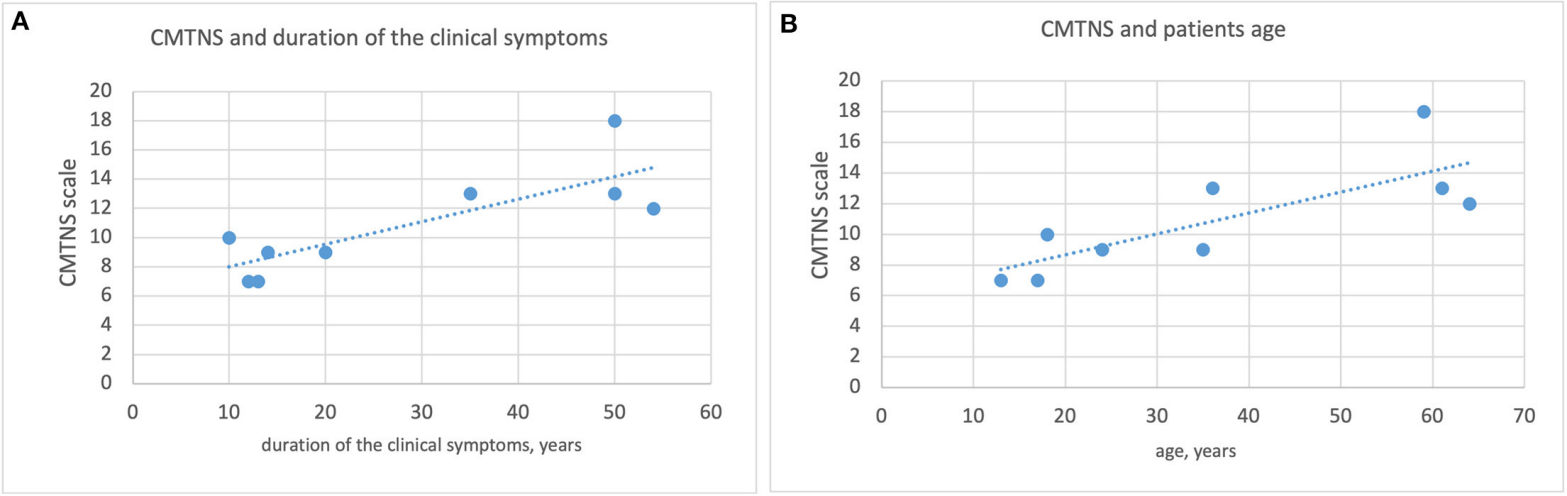
Correlation between the duration of clinical symptoms, patient age and the CMTNS scale. **(A)** Correlation between the duration of clinical symptoms and the CMTNS scale. **(B)** Correlation between pateint age and the CMTNS scale.

A statistically significant correlation was found between the duration of clinical symptoms and the CMTNS scale, as well as between patient age and the CMTNS scale. See Figure 2.

Electrophysiological studies demonstrated axonal polyneuropathy, which was present in 3 out of 10 cases as a pure motor and in 7 out of 10 cases as a motor-and-sensory polyneuropathy. In all cases, nerve conduction velocities were nearly normal, with a severe decrease in motor amplitudes, and concentric needle EMG displayed neuromyotonic discharges characterized by high frequency. Needle EMG also shows increased amplitude of motor unit action potentials. In the patient group with pure motor axonal neuropathy, myotonic symptoms in the legs were more severe, with spastic gait disturbances.

**Figure 3:**
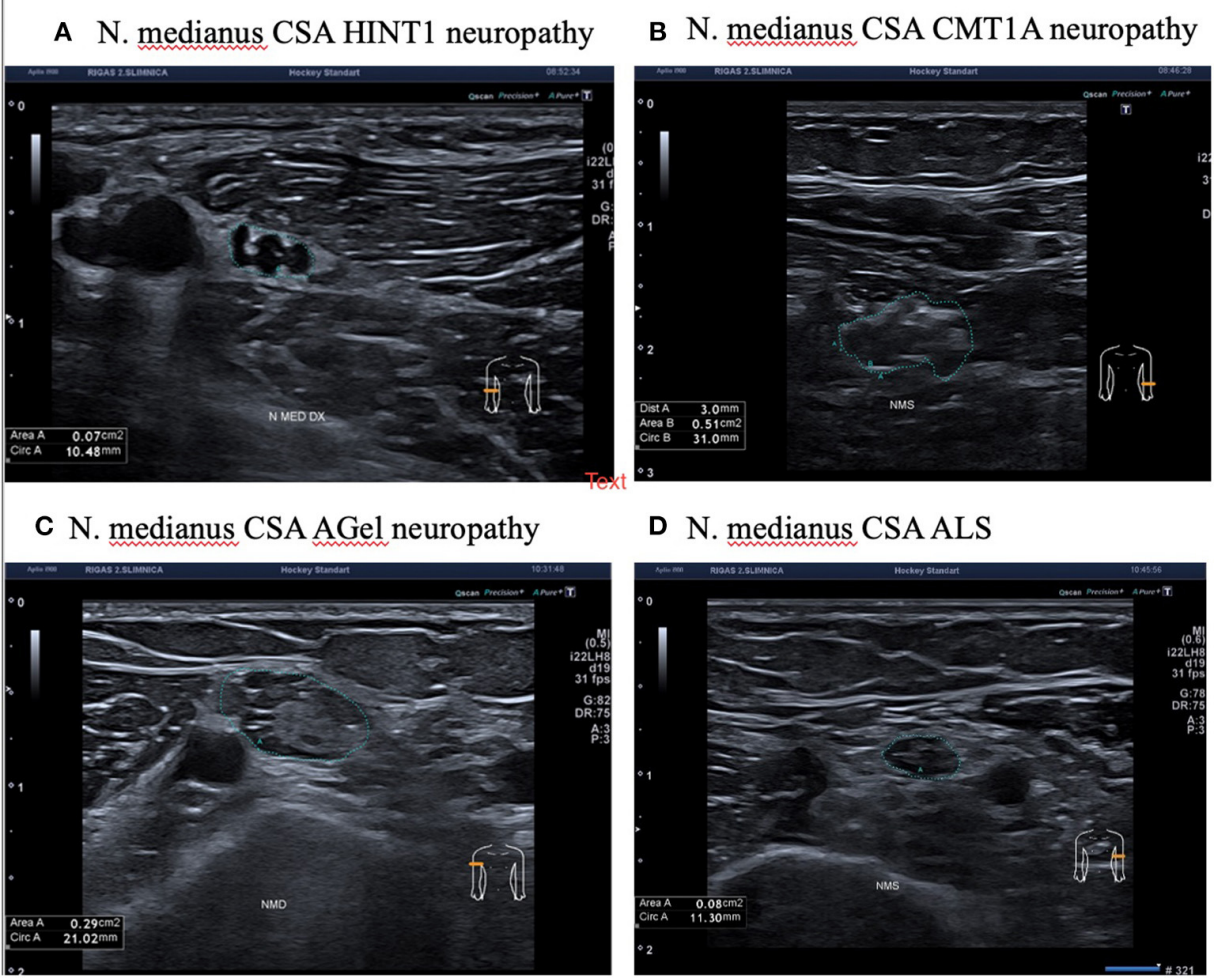
Median nerve ultrasonography in patients with HINT1 neuropathy, CMT1A, ALS, and AGel. **(A)** N. Medianus CSA *HINT1* neuropathy. **(B)** N. Medianus CSA CMT1A neuropathy. **(C)** N. Medianus CSA AGel neuropathy. **(D)** N. Medianus CSA ALS.

Ultrasonography was performed on 4 patients out of 10 who had *HINT1* neuropathy. The nerve cross-sectional areas (CSA) of the median and ulnar nerves were closer to the lower limits of the normal values (11). None of the investigated nerves had structural changes. Compared to patients with *HINT1* neuropathy, CMT1A and AGel patients had enlarged nerve cross-sectional areas and changes in nerve structure. Patient with ALS and axonal changes in a nerve conduction study had similar nerve cross-sectional areas and normal nerve structure (Figure 3).

In all patients with *HINT1* neuropathy, ultrasound examination showed significantly reduced muscle volume as well as spontaneous fasciculations and fibrillations (see Supplementary Table 2). Patients with CMT1A, AGel neuropathies and ALS had similar muscle changes with spontaneous activities. Neuromuscular ultrasonography findings are presented in Supplementary Table 2.

Different psychiatric and cognitive problems were present in six out of ten cases, and six out of nine patients were likely to be diagnosed with anxiety or a related disorder (GAD 7 scale >5). The median GAD 7 scale was 7 points (ranging from 0 to 13), which can be interpreted as moderate anxiety (12). For one patient, the GAD 7 scale was not applicable due to severe behavioral impairment.

## 4. Discussion

Neuromyotonia was present in all reported patients and is thus a diagnostic hallmark. Neuromyotonia is expressed as spontaneous muscular activity at rest (myokymia), impaired muscle relaxation (pseudomyotonia), and contractures of the hands and feet (13) and can be observed with or without overt peripheral neuropathy (14, 15). Neuromyotonia results from spontaneously occurring peripheral nerve discharges, often accentuated by voluntary muscle contraction (16).

The phenotype of *HINT1* neuropathy patients is axonal, motor-greater-than-sensory polyneuropathy. Symptoms appear in the first decade of life. At first, patients complain about muscle stiffness and cramps, in both the hands and the legs.

Various types of skeletal deformities are noted in *HINT1* patients. Foot deformities (pes cavus, pes equinovarus, pes cavovarus, or Achilles tendon shortening) are present in a high proportion of cases (1, 7). Scoliosis is reported in one third of patients (7, 17).

Bulk tissue gene expression of the *HINT1* gene is high in the central nervous system, especially in the frontal cortex and cerebellar hemisphere (18). In four studies, homozygous c.110G>C (p.Arg37Pro) patients had behavioral and developmental problems, delayed language development being the most frequent finding amongst them (17, 19, 20). The behavioral and emotional disorders were non-uniform: only one of four affected persons reported social behavioral alterations (19), and another one had a severe conduct disorder requiring risperidone treatment (20). Despite the lack of a known phenotypic delineation, in our study, delayed language development, behavioral disorders and moderate to severe anxiety were present in patients homozygous for the c.110G>C (p.Arg37Pro) variant. Animal models with complete *HINT1* insufficiency have been developed, and none of them have a neuropathy phenotype. Knockout mouse (HINT1-KO) models showed altered social, aggressive, apathetic, and self-neglect behaviors. It was proposed that HINT1-KO mice might even be affected by schizophrenia-like behaviors (18). Another mouse model was indicative of aberrant mood changes, and *HINT1* was subsequently suggested to be a mood regulator (21). However, a study on HINT1-KO mice tested by the forced swim test produced results which are to a certain degree present in all our patients: “increased emotional arousal in aversive situations” (22). We might speculate that unpleasant stimuli (or those considered negative only by patients) in *HINT1*-affected persons alter emotional perception, which can further influence cognitive processes, resulting in delays in such things as learning and problem solving. Still, this hypothesis requires validation in further studies by specialists in the field.

In some patients, a mild-to-moderate elevation of creatine kinase levels is observed (1, 17), probably related to chronic neurogenic muscle atrophy in combination with neuromyotonia.

Our study is the first detailed ultrasonographic evaluation of patients with *HINT1* axonal polyneuropathy. Many general descriptions of ultrasonography in axonal polyneuropathy support our findings. Craig M. Zadman was the first to compare demyelinating and axonal polyneuropathy ultrasound. His study evaluated the sizes of upper extremity nerves along their length in control subjects and patients with neuropathy. Ulnar nerve cross-sectional areas (NCSA) were measured in the upper extremities of 190 subjects, including 100 with neuropathies and 90 controls. In the axonal polyneuropathy group, only seven out of 36 patients (19%) had nerve enlargement (23). Scheidl et al. (24) discovered a different pattern of enlargement of nerves in the case of axonal neuropathy and demyelinated polyneuropathy. Significantly larger CSA values were found in patients with demyelinating polyneuropathies compared to those with axonal neuropathies (24). In our *HINT1* neuropathy group, none of the investigated nerves had structural changes or enlargement.

In 2011, a study on patients with ALS was conducted (25) using ultrasound, a sensitive method for detecting spontaneous muscle activities. Fasciculations were detected with ultrasound in 98% of patients with ALS, whereas electromyography was positive only in 78% of patients. In our ultrasonography study, fasciculations and fibrillations were found in all our patients with *HINT1* neuromyotonia. Fasciculations, which appeared as slight muscle twitches in the *HINT1* neuropathy group, are a non-specific sign of muscle denervation. Similar spontaneous activities were seen in the group of CMT1A and AGel patients with muscle atrophy (Supplementary Video 1) (4, 26).

*HINT1* neuropathy is mainly diagnosed in individuals of European descent. The best known and most widespread disease-associated variant is p.Arg37Pro, which displays an increasing gradient of distribution from west to east in Europe, with the highest frequency in Latvia. Around 50 families are described as carrying this variant. The p.Arg37Pro carrier frequency in outbred populations living in this geographic area is as high as 1:67–182, making *HINT1* neuropathy one of the most common autosomal recessive disorders in this part of the world (1, 7). Another variant, p.His112Asn, is another founder mutation, with five families reported of Italian, Turkish, Bulgarian and (Portuguese) Roma origin. Finally, the p.Cys84Arg variant is present in a homozygous or compound heterozygous state in four Belgian families. Overall, the genetic epidemiology suggests that *HINT1* neuropathy should be considered in the diagnostic work-up of patients of European descent presenting with axonal CMT.

## Data availability statement

The original contributions presented in the study are included in the article/Supplementary material, further inquiries can be directed to the corresponding author.

## Ethics statement

The studies involving human participants were reviewed and approved by Central Medical Ethics Committee of Latvia (No. 3/18-03-21). Written informed consent to participate in this study was provided by the participants' legal guardian/next of kin.

## Author contributions

MR: article preparation, patients evaluation, and data analysis. BL and VK: article concept, patients evaluation, and data analysis. DR and LG: molecular biology work and data analysis. RL, SM, MT, and VL: nerve and muscle electrophysiological studies and data analysis. DL and IM: patients evaluations and article preparation. All authors contributed to the article and approved the submitted version.
