# Supplementary material for: The most common European HINT1 neuropathy variant phenotype and its case studies
Source: Front Neurol. 2023 Feb 17;14:1084335. doi: 10.3389/fneur.2023.1084335 (PMC9981799; doi:10.3389/fneur.2023.1084335)
Supplement: Supplementary file 1 [file Table_1.pdf]

Supplement to “The most common European HINT1 neuropathy variant phenotype and its case studies”

Supplement Table 1. Allele frequency of variant c.110G>C (p.Arg37Pro) in European populations.

Population	Allele frequency
Latvia	0.0100
Bulgaria	0.0037
Estonia	0.0031
Russia	0.0020
Finland	0.0009
Sweden	0.0005
Northwest Europe	0.0001
South Europe	0.0000

Supplement Table 2. Nerve ultrasonography of patients with *HINT1* neuropathy

	Median nerve cross-section area (CSA) dx (cm^2)								Ulnar nerve cross-section area (CSA) dx (cm^2)							
	P1 HINT1	P2 HINT1	P3 HINT1	P4 HINT1	P5 CMT1A	P6 CMT1A	P7 AGel	P8 ALS	P1 HINT1	P2 HINT1	P3 HINT1	P4 HINT1	P5 CMT1A	P6 CMT1A	P7 AGel	P8 ALS
1	0.07	0.09	0.10	0.09	0.08	0.19	0.15	0.11	0.06	0.04	0.04.	0.05	0.06	0.10	0.05	0.06
2	0.06	0.04	0.06	0.06	0.18	0.23	0.11	0.09	0.04	0.05	0.07	0.05	0.07	0.15	0.06	0.06
3	0.06	0.05	0.11	0.05	0.27	0.38	0.11	0.07	0.05	0.06	0.06	0.05	0.14	0.25	0.08	0.11
4	0.07	0.06	0.11	0.08	0.12	0.32	0.16	0.10	0.07	0/06	0.07	0.08	0.08	0.13	0.14	0.08
5	0.10	0.05	0.10	0.09	0.58	0.33	0.16	0.20	0.06	0.06	0.06	0.04.	0.17	0.25	0.14	0.12
6	0.10	0.05	0.07	0.06	0.44	0.28	0.29	0.11	0.05	0.05	0.07	0.08	0.32	0.33	0.21	0.11

Median nerve positions: 1) on the level of the carpal canal, 2) proximal to the carpal canal, 3) middle 1/3 of the forearm, 4) proximal to cubital fossa, 5) in the cubital fossa, 6) on the level of armpit region (axillar region);

Ulnar nerve positions: 1) on the level of the Guyons canal, 2) proximal to the Guyons canal, 3) middle 1/3 of the forearm, 4) below elbow 6) above elbow
